# Supplementary material for: Sodium, Potassium-Adenosine Triphosphatase as a Potential Target of the Anti-Tuberculosis Agents, Clofazimine and Bedaquiline
Source: Int J Mol Sci. 2024 Dec 4;25(23):13022. doi: 10.3390/ijms252313022 (PMC11640857; doi:10.3390/ijms252313022)
Supplement: Supplementary file 1 [file ijms-25-13022-s001.zip › ijms-3237044-supplementary.pdf]

List of figures in supplementary

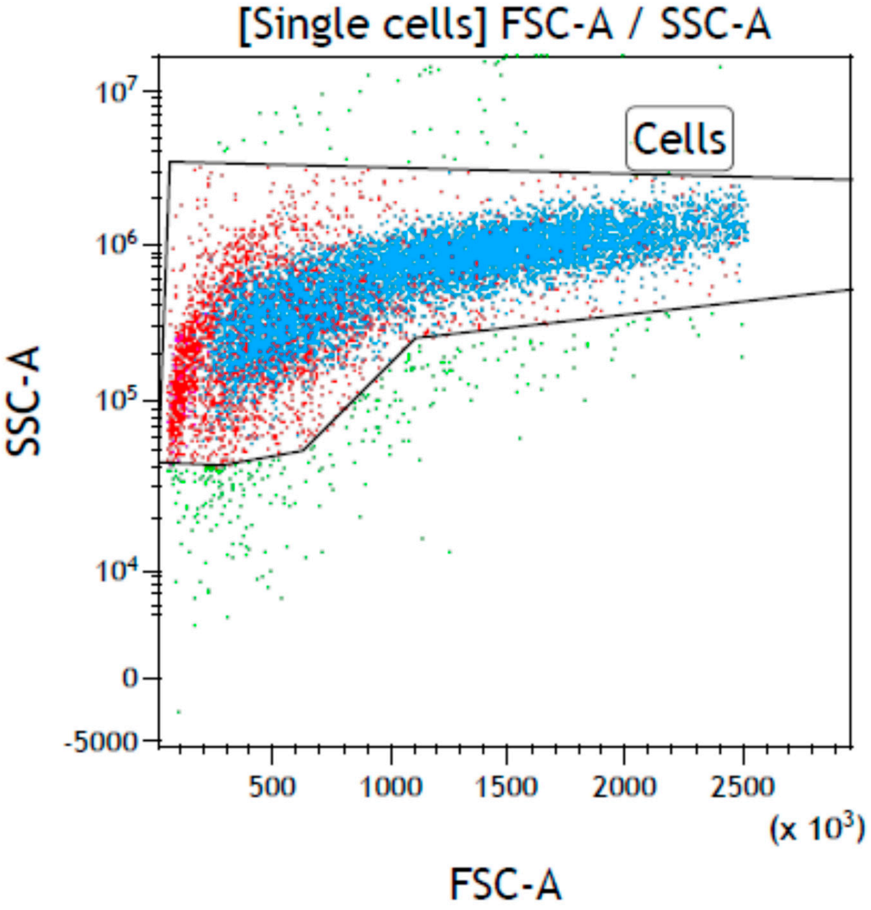

Figure S1: Flow cytometric scatter plot demonstrating the size of the rat cardiomyocytes (RCMs). The RCMs are large cells, with most of them being positioned between 10^5 and 10^6 coordinates on the Y-axis and zero and 500 on the X-axis. The majority of the cells are positioned closer to zero on the X-axis showing that they are viable (red dots).

Abbreviations: FSC, forward scatter; SSC, short scatter.

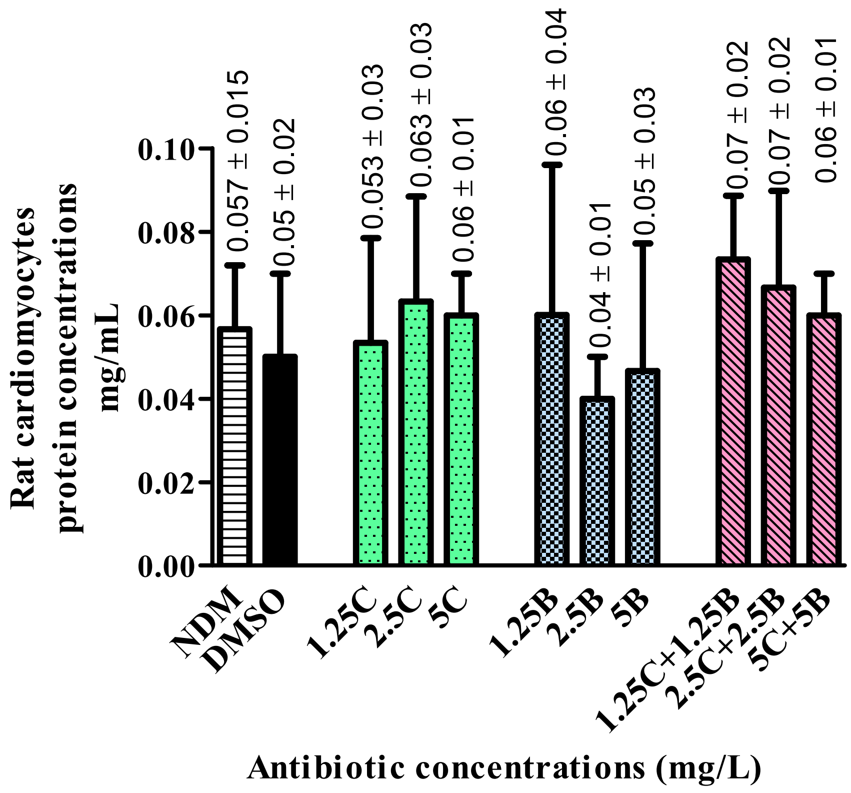

Figure S2: The protein concentrations (mg/mL) of antibiotic-free and antibiotic-treated RCMs, extracted using vortex methods, which were used for the Na^+,K^+-ATPase activity determination, measured using nanodrop mg/mL. Abbreviations: B, bedaquiline; C, clofazimine; DMSO, dimethylsulphoxide; NDM, no DMSO.

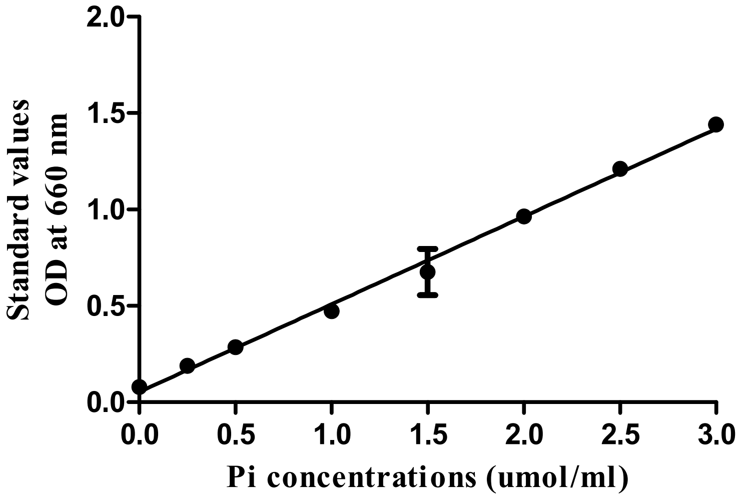

Figure S3: The standard curve for inorganic phosphate (Pi) concentrations. The slope of the graph was Y = ax + b with a = 0.4551 ± 0.01140, while b = 0.05283 ± 0.01926.

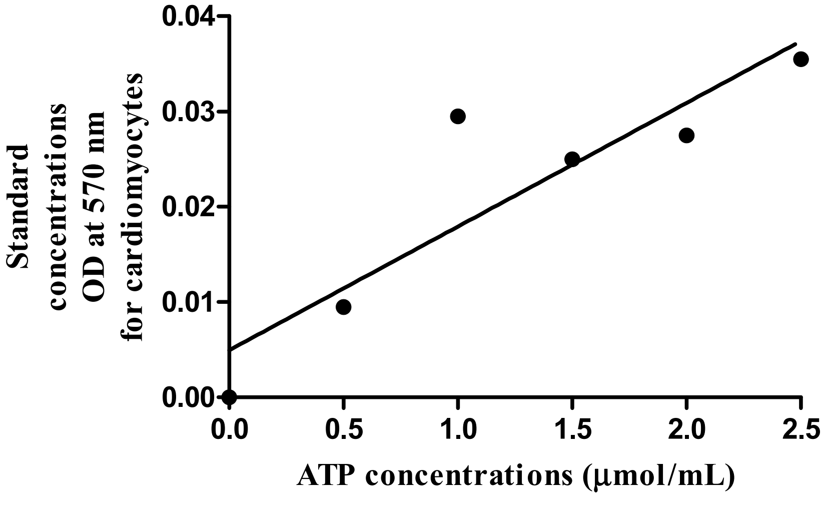

Figure S4: The standard curve for determination of adenosine triphosphate (ATP) concentrations. The slope of the standard curve was y = ax + b = 0.013 ± 0.003x + 0.005 ± 0.0048. R^2 = 0.8055

Supplementary tables

Table S1: The variables used for determination of the Na^+,K^+-ATPase activity formula

Variable	Description	Detail
ΔA_{660}	$OD_{sample} – OD_{control}$	
C_{pr}	Concentration of protein in sample (mg protein/mL)	Figure S2, Supplementary Material
b	Y intercept of standard curve	0.05283 ± 0.01926 (Figure S3, Supplementary Materials)
a	The slope of the standard curve	0.4551 ± 0.01140 (Figure S3)
V_1	The total volume of reaction system	0.25 mL
V_2	The volume of added sample	0.1 mL
t	The time of enzymatic reaction	(1/6 hours, i.e 10 minutes)
f	Dilution factor of supernatant sample before tested	Undiluted
